# GP perspectives on hospital discharge letters: an interview and focus group study

**DOI:** 10.3399/bjgpopen20X101031

**Published:** 2020-05-13

**Authors:** Katharine Weetman, Jeremy Dale, Rachel Spencer, Emma Scott, Stephanie Schnurr

**Affiliations:** 1 Unit of Academic Primary Care, University of Warwick, Warwick Medical School, Coventry, UK; 2 Centre for Applied Linguistics, University of Warwick, Coventry, UK

**Keywords:** communication, hospital discharge, general practitioners, patient discharge, copy letters, doctor and patient communication, discharge summaries, primary care

## Abstract

**Background:**

Written discharge communication following inpatient or outpatient clinic discharge is essential for communicating information to the GP, but GPs’ opinions on discharge communication are seldom sought. Patients are sometimes copied into this communication, but the reasons for this variation, and the resultant effects, remain unclear.

**Aim:**

To explore GP perspectives on how discharge letters can be improved in order to enhance patient outcomes.

**Design & setting:**

The study used narrative interviews with 26 GPs from 13 GP practices within the West Midlands, England.

**Method:**

Interviews were transcribed and data were analysed using corpus linguistics (CL) techniques.

**Results:**

Elements pivotal to a successful letter were: diagnosis, appropriate follow-up plan, medication changes and reasons, clinical summary, investigations and/or procedures and outcomes, and what information has been given to the patient. GPs supported patients receiving discharge letters and expounded a number of benefits of this practice; for example, increased patient autonomy. Nevertheless, GPs felt that if patients are to receive direct discharge letter copies, modifications such as use of lay language and avoidance of acronyms may be required to increase patient understanding.

**Conclusion:**

GPs reported that discharge letters frequently lacked content items they assessed to be important; GPs highlighted that this can have subsequent ramifications on resources and patient experiences. Templates should be devised that put discharge letter elements assessed to be important by GPs to the forefront. Future research needs to consider other perspectives on letter content, particularly those of patients.

## How this fits in

This study builds on previous evidence through exploring GP views on discharge letters. Interviews and focus groups allowed in depth understanding of GP views on patients receiving letters and the content of successful discharge letters. There remains a need to improve written discharge communications, and the findings indicate ways in which this could contribute to improved patient experience.

## Introduction

In the UK, 'discharge communication' may follow inpatient discharge or outpatient clinic discharge from hospital; it typically comprises written communication that is sent to the GP. Hospital admissions often involve complex changes to patients' diagnoses and medications; without adequate communication to the GP, care transition would carry unacceptable risks.^1^ Regulatory bodies already recommend that patients receive a copy of their discharge summary.^2–7^ National^8^ and international^9^ standards for discharge communication are stipulated, yet despite this, studies,^10–17^ both within and outside the UK, continue to reiterate that processes and content of discharge communication require improvement. GPs have previously been found to have a range of opinions from acceptance to active rebuttal on delegation of workload requested in discharge summaries.^18^ It is known that poor-quality discharge communications can lead to adverse outcomes,^15^ such as preventable readmissions to hospital.^19^ The authors' previous work shows that patient safety can be compromised by the management of discharge summaries in primary care^20^ and that the design of the discharge summary is a key element of these safety issues. Further study is needed to build on recommendations from this work.^18^

Copying patients into NHS letters is currently 'good practice'^2–7^ but not standardised. The result is that patients are inconsistently copied into discharge communications,^21,22^ but the reasons and effects of this remain unclear.^23,24^ While GPs generally favour patients receiving letters,^25,26^ some reservations, such as concern regarding patient comprehension of medical terminology, have been expressed.^26–28^ However, much of the evidence on GPs’ perspectives is from over 10 years ago, and little is known about current GP perspectives on patients receiving discharge letters and how this should be undertaken to improve patient experiences and outcomes.^24^ It is important to understand the effects of copy letters on patient outcomes owing to the associated costs of this practice and the potential for negative effects.^26–28^ A recent review by Harris *et al* in 2018^23^ expounded that further research is needed to understand the impact of copying letters to patients on health outcomes as well as the content and accuracy of copy letters. Hence, the objectives of this study were to gather GP perspectives on discharge summary content and on copying patients into discharge letters. The research questions were:

- Research question 1: according to GPs, what content items do successful discharge letters contain?
- Research question 2: should patients receive discharge letters, why and in what form?

## Method

### Recruitment and data collection

This study opened in August 2017 and involved recruiting GP practices within the West Midlands (England, UK). Practices were invited to take part through invitations circulated via the local primary care clinical research network (CRN) team, collaborating clinical commissioning groups (CCGs), and university links with practices. All GPs at participating practices were eligible to take part; individual GPs expressed interest through direct email, discussion during site visits, and communications via the CRN.

This study is one of three complementary studies forming the Discharge Communication Study (see published protocol ^29^) that sought to explore how patients receiving discharge letters works as an intervention. The first comprised two parts and involved 53 GPs selecting and commenting on a sample of discharge letters; a content analysis of the letters was completed alongside comment analysis. The second part of the first study is the focus of this article, which is an analysis of interviews and focus groups with the GPs who had participated in the first study (recruitment target 90%). The second study involved interviews with patients and the third was formed of a professional survey at hospitals (for further details see the Discharge Communication Study protocol ^29^).

GPs were invited to take part in an interview or focus group which, for convenience, could take place face to face or over the telephone. The purpose of offering both was to allow GPs a choice of whether they would prefer to discuss their views alongside their colleagues or just with the first author. It was anticipated that the focus group option would allow data collection to take place during existing GP practice meetings and so allow increased participation. Interviews and focus groups were 'narrative'^30^ with an opening question around GP experiences of discharge letters (see Supplementary Box S1 for focus group and interview schedule). Interviews and focus groups were audio-recorded. The running time was flexible and most ran for 20–30 minutes as anticipated, creating around 12 hours recording time.

### Data analysis

CL is the study of language through corpora,^31^ which are electronic, machine readable 'collections of texts'.^32^ CL is a branch of linguistics that focuses on analysing patterns of co-occurrence and meanings in corpus data^33,34^; its application can bring new insights to research questions.^35^ CL has previously been demonstrated as useful for health-related research related to communication^36–40^ and, therefore, was considered suitable for this study. CL techniques allowed pattern-identification of important content items (research question 1) and GP views on patients receiving letters (research question 2).

Interview and focus group data were analysed in the same way. Interviews were narrative^30^ and so interviewer prompts were minimal and removed from text files in order to focus the corpus on GP views only. Focus group data contributions were separated and subsequently labelled by the contributor for corpus consistency of one text file per participant. The result was that each text file comprised a single GP participant viewpoint. It is acknowledged that disentangling the focus group may have lost meaning in parts of dialogue where participants conceptualised ideas across multiple turns.

Participant response data were transcribed, and consolidated in Antconc^41^ — which is a freeware corpus analysis toolkit — to build the corpus. Quantitative techniques in the form of keyword lists^31^ were used as the point of departure for identifying salient linguistic features.^42^ A 'keyword list' is a ranked list of 'keywords' generated when the frequency lists of two corpora are compared statistically^43^. 'Keywords' are words that are statistically more significantly frequent^31^ or 'key' in one corpus list compared with another.^43^ As Szudarski^43^ explains, keywords are useful in providing 'information about the keyness or specificity of a given corpus in terms of what it is about'^43^. Following Baker *et al*,^44^ the statistical calculation for sorting 'keywords' by 'keyness' was log-likelihood (LL). This was set to the Antconc^41^ default settings (5% level; *P*<0.05). The British National Corpus (BNC) Spoken (2014)^45^ was used as a reference corpus for generation of keywords owing to the comparable spoken data mode. Thereafter, qualitative techniques, informed by the quantitative findings, were used.

The GP data had no inherent categorisation system as interviews were narrative. Therefore, following examples of previous corpus studies^38,40^ dealing with somewhat unstructured bodies of data, keyword results were put into 'themes'. A 'theme' was to be formed of grouping keywords by their contextual meanings that were salient to the research questions. Themes were defined initially by the first author during preliminary qualitative analyses of keywords through concordance line reading and re-reading, and then confirmed by other members of the research team. The use of 'themes' here to group keywords and structure the write-up of analysis is distinct from a 'thematic analysis',^46^ whereby the data would be read, coded and then checked for theme validity.^46,47^

Thereafter, qualitative techniques to investigate and examine collocation^48,49^ and concordance lines^31^ were undertaken; this allowed more in depth exploration of quantitative findings. 'Collocation'^34,49^ is the phenomenon whereby words or 'collocates'^48^ habitually co-occur with one another.^50^ Collocates can be reckoned through statistical calculations such as LL or mutual information.^51,52^ 'Concordance lines'^31^ display words of interest, 'keywords' or 'nodes' in their context within the text, that is, with a chunk of co-text either side^43^; the standard display for concordances is termed keyword in context (KWIC) lines.^50,53^ Concordance lines permit rapid identification of language patterns.^54^ All concordance line outputs were read and random samples were used to illustrate patterns; identification codes of transcripts for sample lines were checked to ensure the samples were not drawn from the same one or few participants. The number of characters displayed either side of the keywords in the concordance lines was automatically generated using the Antconc^41^ software (characters = 25 either side of the keyword); this character count does not include spaces and lines were tidied to ensure words were not cut-off.

Additionally, dispersion^55^ of patterns was considered. Dispersion refers to how evenly or unevenly a word is spread across the corpus.^34,55,56^ Observing distributional patterns through considerations of dispersion were made utilising the Antconc^41^ 'concordance plot' function. This function displays: the overall number of word occurrences, the number of texts in which the word occurs, and the number of times the word occurs within each text. This can help avoid or at least reduce false positives and over-generalisations.^57^ Transcripts were also hand-searched for any 'missed' patterns.

## Results

### Participants

A total of 26 participants (49% of the 53 who were eligible to participate) from 13 practices were recruited. This included 20 individual interviews and one focus group with six participants. Recruited practices included one small practice (<5000 registered patients), seven medium practices (5–10 000 registered patients), and five large practices (>10 000 registered patients). The practices were spread across a range of urban and rural areas in the West Midlands with varying sociodemographic characteristics. For example, several of the participants worked in GP practices in South Warwickshire, an area with a predominantly white ethnic population^58^ whereas Coventry has a more ethnically diverse population with a large black and minority ethnic population compared with the national average and other sub-regions within the research.^59^ The diversity of residents within these localities meant that the patient registries of participating practices also exhibited diversity. Hence, this increased the variety of discharge communication experiences captured in the study.

Participants ranged in age (28–59 years, median = 43) and experience. Less variation was seen with regards to ethnic group (white British 78%). There were 15 female (57.7%) and 11 male participants (42.3%).

### Corpus linguistics

The corpus contained 26 text files categorised by participant (*n* = 26) and contained 53 643 words^50^ and 2828 word types.^50^ The top 100 keywords are in Supplementary Table S1 with concordances in Supplementary Box S2. Keywords of interest to the research questions were grouped into 'themes' seen in Table 1. The results are presented by theme.

**Table 1.** Keyword grouping themes relevant to research questions

**Theme**	**Research question relevance**	**Keywords**
Discharge letter content items	Research question 1	follow (up), medication(s), drugs, investigations, tests, diagnosis(es), admission, renal, CT, results, plan, action, acronyms, blood(s), medical, appointment, clinical, information, discharge(d), hospital, GP(s)
Patients receiving discharge letters	Research question 2	Patient(s), cop(ies), communication
Discharge letters forms/types	Research question 2	Summary(ies), template, outpatient, written, handwritten, letter(s), clinic
Practitioners writing discharge letters	Research question 2	doctor(s)

CT = computed tomography

### Discharge letter content items

Positive evaluative adjectives (for example, 'clear' *n* = 111) framed the following content items as important to the participants: diagnosis, GP actions, pending investigations, medication, follow-up plan, patient information, and clinical summary to include reason for admission and what occurred in hospital.

A 'follow-up' plan (*n* = 80) was often talked about as a 'necessity'; participants asserted that they needed to know 'what' the follow-up plan is in addition to information regarding the agent ('who') is responsible. They caveated that the agent needs to be appropriate to the actions; participants commented that they are not responsible for following up pending hospital results; for example, *'They ought to follow through that one themselves'.* Some participants drew on and coined idiomatic metaphors to stress the safety implications and importance of 'follow-up'; for example, *'Some people do get seen but some go through the net*'. Participants reported that the person delegated for the follow-up plans should be appropriate and in accordance with guidelines and standards (for example, General Medical Council [GMC]*,* NHS England, and Academy of Medical Royal Colleges [AOMRC]).^60^ For instance, issues were expressed about the pragmatics and safety implications of imminent test requests, typically within 1 week of discharge; for example, *'unrealistic', 'the blood tests are not going to get done'*. The GP may have not received the letter before the tests are due and difficulties remain within primary care of providing a rapid blood test service.

Participants favoured the content item box 'GP action'; although, they did note ambiguity issues when the box contained unclear 'actions' or is left 'blank'; for example, *'Presumably there is no action needed it would be much better I think rather than leaving a bit blank to actually say no action required.'*

Participants described having information regarding 'medications' (*n* = 127) and 'drugs' (*n* = 40) 'changed or stopped/started' and reasoning for changes was 'useful' or 'helpful'; for example, *'Information about medication is very helpful whether it is new or started or stopped.'.*

'Investigation(s)' (*n* = 42) and 'test(s)' (*n* = 54) had semantic preference for assessment outcomes; for example, 'results'. Participants favoured detail relating to: what tests have been run and the results, and information regarding pending tests and results.^60^ There was a heightened need for clear results in the letter as GPs cannot necessarily access these, depending on the practice locality and care record operating system. Participants highlighted that clear information can avoid unnecessary test duplication and hence preserve resources.

Participants indicated diagnostic information (*n* = 106) is a requisite for discharge communication. Letters with a clear and accurate diagnosis were judged or conceptualised as successful; for example, *'A successful discharge letter in that the diagnosis was clear.'* Participants found it useful when the letter clarified the patient’s diagnosis knowledge. Some participants contemplated whether medical terminology to elucidate diagnoses may need lay explanations for patients. One participant suggested that use of medical terms only can be alienating for patients and *'reinforces the fact that medicine is scientific and complicated and you have got to be clever to understand it …*' References to 'acronym' (*n* = 27) were frequently negative; this was evidenced through co-occurring words with negative connotations (for example, 'AVOID' [lines 2, 4, 10], 'bad' [line 1], 'wrong' [line 6]) (see Table 2); Participants suggested acronyms should be avoided both for the sake of the patient and themselves (lines 7–10).

**Table 2.** Sample of 10 random concordance lines for 'acronym'

Line 1	from [LOCATION] but yeah I mean	**acronyms**	are generally a bad thing aren’t they (.)
Line 2	yeah ideally you want to be avoiding	**acronyms**	as far as possible um and particularly
Line 3	and it was CRT there are quite a few	**acronyms**	in there but I think I understand those
Line 4	be more clearly set out well I think	**acronyms**	should be avoided wherever possible
Line 5	of typing and I know there are some	**acronyms**	that could be applied to more than
Line 6	a bar in the occasional letter or an	**acronyms**	that has gone wrong or mistakes that
Line 7	we don’t always understand the	**acronyms**	that they use and I think on a formal
Line	with date is all on there um (.) one	**acronym**	I don’t know myself so not sure how
Line 9	here or could you clarify what this	**acronym**	stands for because we are not always
Line 10	large they ought to avoid using any	**acronyms**	really for clarity not just to the patient

Letters described as 'successful' (*n* = 45) were an appropriate length, *‘* *not too wordy* *’* and contained a clear and accurate diagnosis. Lines for 'information' (*n* = 184) conveyed it is not just the presence of information that governs a 'successful' letter but the information quality. However, participants did remark on the difficultly of balancing the right level of information and the need to personalise and adapt information for different cases.

### Patients receiving discharge letters

Participants noted inconsistency of 'patient(s)' (*n* = 704) receiving discharge letters:

*'… sometimes patients will get a copy of that and sometimes they won’t.*'

*'… within hospitals and even within departments and even different consultants some choosing to share information with patients and others not so there doesn’t seem to be any unified approach to it.'*

​*'My experience of patients receiving written discharge is that it is variable and does not always happen*.'

One participant suggested a patient copy receipt 'click box' for clinicians writing summaries could resolve uncertainties and monitor inconsistencies through auditing. The majority of participants (85%, 22/26) indicated that they felt giving letters to patients is considered to be a good idea; for example, *'Generally I think it is an excellent idea that we give patients their discharge summaries.*' This was amplified by surrounding positive evaluative adjectives: *'good', 'useful',* and *'handy*'. Participants noted the patient's right to the letter, *'I think it is theirs*'. Favourability was seen even in cases where the practice had not taken place:

*​'It would be entirely appropriate for the patient to see a letter of that nature but I notice the consultant hasn’t copied it to the patient.'*

One participant recounted a recent anecdote where a patient having the letter remedied a situation where the GP had not and thus saved time:

*​'I just had a patient … and she has been in hospital with … this and that and the other and the discharge letter hadn’t come to us and luckily she had one and brought it in.'*

Quotations where participants indicated giving letters to patients is a good idea were often accompanied by highlighting of positive outcomes. The described outcomes included: a sense of patient inclusion, increased patient understanding, patient autonomy, enhanced communication transparency, and that the letter can act as a memory-aid (for example, medication). Participants highlighted that the paper-held summary may also act as a physical record of the admission for future encounters and communications, particularly if the patient sees a team who do not have access to the letter (for example, out-of-hours GP).

Participants were not uniformly in favour of patients receiving letters in their current proforma:

*'The discharge letter is there as a succinct conveyance of information, it is not a patient education letter as such so if you were to try and take every bit of medical jargon out then it becomes a patient education letter, which is then going to become less useful for the clinician because you are going to have to wade through a lot of excess information.'*

Negative outcomes or issues noted by participants were said to include difficulties of producing a letter suitable for the needs of the GP and the patient; for example, *'... but it’s just I suppose the information that we need is often different from the information that the patients need in terms of just thought processes*'. They also included patient confusion; for example*, '… but then it can be tricky with a lot of the clinical information whether that might cause them more concerns if they don’t understand necessarily everything that has been written.'* Other negative outcomes or issues were: letter inaccuracies alarming patients, language barriers and patient low literacy raising health inequalities, increased patient anxiety or harm, GP workload to explain letters to patients, ethics of cases where the diagnosis had not been disclosed, confidentiality breaching if the letter contained third-party information or if the patient misplaced the letter, and the patient upset in relation to sensitive issues (for example, obesity). Participants suggested modifications to address these issues:

1. Considerations of patient choice; for example, *'… as they were given the choice of whether to take it or not then I can’t see that that would be a problem ...*'
2. Giving the patient an abbreviated form; for example, *'So maybe a clearer copy not necessarily the same as the copy that the GP gets but a more abbreviated form for the patient ...*'
3. Simple interpretations of results; for example, *'… it could have some sort of explanation after all those numbers to say these are all normal or these are all satisfactory.*'
4. Inserting a patient information section; for example, *'I think a box at the bottom that says information for the patient and then a line with a layman’s explanation about it.*'
5. Using plain English and explaining jargon with lay terms; for example, '… *lap right hemicolectomy just to put bowel surgery*.'
6. Adding a parallel patient box; for example, *'… there should also be a very clear section saying … what the patient should be doing …*'

Participants considered verbal advice to be pivotal to patient understanding and argued that the letter should not be the sole information source; for example, *'* *I* *t is one piece of patient information not the only piece* *.*' One participant summarised, '…*if patients are not understanding their discharge letter it’s because they have not had the counselling to go with that discharge letter.*'

One participant’s discourse featured commentary about the importance of communication to patient care and this GP also pondered the potential benefits of policies in this area:

*​'We probably generally have too many policies for too many different things but I think communication if it’s not being done well probably is something that needs a policy so that it can be adhered to and audited and checked up on.'*

### Discharge letter forms or types

Across lines, patterns emerged for 'handwritten' (*n* = 21) forms of communication to have issues with illegibility and clearness: *'I don’t know what the hell it says', 'often it’s illegible*'. Furthermore, 'handwritten' forms were talked about as a thing of the past; for example, *'… they are a bit behind on the game still sending out handwritten notes …*' or something to be 'avoided'; for example, *'… avoiding handwritten communication certainly helps.*' Contrastingly, typed 'template' (*n* = 21) forms often co-occurred with positive evaluative adjectives; for example, *'I think the template here is a good template', 'I quite like the template ones if I’m honest they are better …*' Nonetheless, two participants did suggest the template could impose limitations or *'[fall] down*'.

A few participants reflected on whether the discharge letter template is designed around the informational needs of the GP or the patient or both. One explicitly outlined issues with producing *'two different discharge summaries*' (that is, one for the patient and one for the GP) and argued the benefits and drawbacks of the patient receiving the 'same' as the GP. These arguments appeared to align with the findings for patient letters and copies described above:

*'I think the (.) it would be a lot of work for them to do two different discharge summaries and then the risk of things being left off or omitted so I think practically its probably helpful for the patient to have the same letter as we have but I think the danger of that then is that there is a lot of technical stuff in there.'*

### Practitioners writing discharge letters

'Doctor(s)' had 67 hits in the corpus across 14/26 texts; this is noticeably less hits than that of GPs (*n* = 177) and patients (*n* = 704). Participants raised trepidations around junior doctors writing discharge letters given their experience (examples in Table 3, lines 11–15).

**Table 3.** Illustrative concordance line samples regarding concerns with juniors writing letters

Line 11	sometimes it is because brand new	**doctors**	are the ones writing the letters and they are
Line 12	better than the medical bit by junior	**doctors**	(.) some junior doctors do extremely well but
Line 13	aren’t clear cut and junior hospital	**doctors**	don’t always necessarily have a really good
Line 14	hospital doctors particularly junior	**doctors**	haven’t really learnt that skill of putting things
Line 15	allowed to say that anymore junior	**doctor**	who understands what we need and

Particular concerns were voiced in relation to junior doctors and otherwise, where the patient may not be known to the doctor writing the letter:

'… *so it’s always ominous when it says discharge summary completed from notes patient not known to doctor so immediately that tells us (.) that the poor junior doctor writing the letter has never seen the patient so that never bodes well in terms of accuracy of the report.'*

Two lines expressed difficulties when the letter author cannot be identified; for example, *'It’s not clear who the doctor was …*'. One participant suggested a potential solution to issues with juniors writing letters in that the letter does not necessarily need to be produced by a doctor; they specified the individual, *'just needs to be somebody who has enough of a grip of what changed and what is planned*' and so could be a ward clerk, nurse and so on. Another participant contextualised the conflicting demands on GPs and hospital doctors and the impact of this on communications; they suggested junior doctors may be ignorant of general practice systems, which can cause issues with action requests:

*​'… they have never been in general practice and they think it is just easy to just to get a blood test done like it is in hospital because you put a form out and the bloods get taken like magic and that is a bit frustrating...'*

**Table 4.** Summary of main findings across results and analyses for all themes

**Main findings**
Key content items associated with successful discharge letters A clear diagnosis. Appropriate follow-up plans with clear agency. Medication information, including any changes and reasons. Avoidance of acronyms. No 'blank' boxes on templates. Views about copying patients into discharge communication In general, patients should be offered a copy of their discharge letter. Patients need to receive adequate verbal advice alongside written information. Not all patients want letters and so considerations of patient choice are important. Addition of a patient information section may make letters more useful for patients.

## Discussion

### Summary

This study found a broad consensus on GPs’ views about the content of 'successful' discharge letters. GPs generally favoured patients receiving discharge letters but felt that some modifications may be required to increase patient understanding and letter usefulness. GPs made several recommendations for improving practices (see Table 4).

### Strengths and limitations

A key strength of this study is that to the best of the author's knowledge, this is the first study to apply CL methods to UK qualitative data on GP experiences of discharge communications. A linguistic approach to the data facilitated an analysis not just of 'what' was said but 'how' it was said. This allowed detailed consideration of how discharge communication was described and evaluated by the GPs.

The findings provide valuable insights into GPs’ views and experiences related to the research questions. The study recruited 26 GPs (49%), which was below the recruitment target of 90%; reasons for this may have been related to insufficient study incentives and competing demands on GPs’ time. The sample was not intended to be representative of GPs in the UK; the purpose of the research was to draw out issues surrounding discharge communication from the perspective of GPs. Participants varied in their age and experience and worked in a varied range of areas and practices (large and small), and the sample had experience of discharge summaries from at least four hospital trusts. However, medium-sized practices were slightly under-represented in the response sample compared with the invited sample. Additionally, semi-urban areas were over-represented in the response sample whereas rural and urban areas were under-represented.

There were 15 female (58%) and 11 (42%) male participants in the sample. This skew towards female GPs is not representative of the registered NHS GP population, which was reported by the GMC in 2018^61^ to be far more balanced: male GPs 51%, female GPs 49%. Reasons why more females participated in the study were unclear.

For this study, the standards for reporting qualitative research were followed.^62^ One of the strengths of the CL methodology is that it allows manual and computer-assisted study of more generalisable quantitative features as well as fine-grain qualitative analyses.^40^ The computer-assisted features of modern corpora mean that CL software has the potential to search, sort and process data with speed and replicability.^63,64^ Corpus processing can reveal language patterns and commonalities as well as rare cases; neither of which are likely to be available solely through intuition.^48,51^ Consequently, CL permitted rapid identification and exploration of patterns and thus analysis of a larger body of data than would have been feasible through manual searching alone^31,40,56^ . Considerations of dispersion reduced but did not eradicate bias that may have resulted from interview length variability. As McEnery and Hardie^65^ suggest, the quantitative nature of CL increases accountability and replicability of findings. However, it must be acknowledged that the analysis cannot be considered fully objective or unbiased owing to the influence of researcher identity^66^ and unconscious bias.

### Comparison with existing literature

The mixed-methods approach has generated findings that are both broad and generalisable to an extent, such as letter components important to GPs, and also in-depth viewpoints on current processes of discharge communication and how they may be improved.

Content features framed by participating GPs as important to successful discharge letters supported findings from previous literature^67,68^ and published guidance.^60,69^ However, junior doctors may not be receiving the quality training they need to produce satisfactory discharge letters as evidenced both by the concerns of the GPs in this study, and another,^18^ and by a previous study with junior doctors.^70^

GP-deemed beneficial outcomes of patients receiving letter copies in the data resonated with previous studies.^22,25,26^ As in past research,^26–28^ the study sample GPs had concerns regarding patient understanding and explained that discharge letters do inherently need to contain a degree of scientific and technical medical information for the GP, which may not be comprehensible to a patient. Additional issues raised were: cases where the patient does not yet know the diagnosis or does not want to know the diagnosis, cases where the letter content may cause the patient harm, and letters that contain information for which the patient does not have permissions to view (for example, third-party information). Participating GP suggestions to address these concerns and issues resonated with previous studies^2,22,71–73^ and included: avoidance of 'blanket' copying letters, inclusion of a short patient information or action section on letter which clarifies relevant information (for example, 'blood in urine' instead of 'haematuria'), providing patient with abbreviated form, explanations of jargon with lay terms, use of plain succinct English (for example, short simple sentences), and interpreting test or procedure results into simple 'patient friendly' terms (for example, all blood tests normal). Additionally, results revealed that unexplained acronyms should be avoided within discharge letters, for comprehensibility both to the patient and GP; the data here indicated that misinterpretations as a result of acronyms in written communications can be time-consuming to remedy. These modifications and suggestions align with the Professional Record Standards Body records guidance^74^ as well as the *'Copying letters to patients: good practice guidelines'* by the Department of Health^2^ and the more recent 'please write to me' initiative by the AOMRC.^4^

### Implications for research and practice

The findings have relevance to all developed nations with similar transition points in the patient journey between secondary and primary care. Practical recommendations have been made that could be introduced in many healthcare systems. A caveat is placed on this: the cultural and healthcare climate might affect nuances of stakeholder opinion and further study in other nations would enhance the evidence base.

Despite copying patients into letters sent between physicians being considered to be good practice in the UK,^2^ GPs reported that patients are receiving letters inconsistently. This indicates a continuing need for standardising policy and hospital trust procedures as opposed to merely making recommendations. However, some patients may not want to receive letters or there may be risks of harm and so patient choice and considerations of the individual case are paramount. It is suggested that if all policies and guidelines^2–5,23^ regarding patients receiving letters were consistently adhered to, few issues would remain. Hence, work is needed to increase the uptake of policies and guidelines and overcome implementation barriers. Future work should outline the cost benefits of this practice.

Broadly, GPs felt that it is possible for letters to be 'patient friendly' while at the same time meeting the needs of general practice. Thus, perhaps there is no need for two separate summaries. Sending patients direct copies not only adheres to good practice but also may save time and resources,^2,21,73,75^ and fits with the broader policy goals of shared-decision making, transparency, and patient-centred care.^76,77^

GPs reported that letters frequently lacked content items they assessed to be important to successful letters; GPs highlighted that this can have subsequent ramifications on resources and patient outcomes. Participating GPs commented that where headings or components in template discharge communications are not relevant to a particular episode of care, it should be explicitly stated for increased clarity (for example, no action for GP) rather than left blank. This could be remedied with a computer system that mandates text in each template box.

Overly long letters were often portrayed as verbose and clouding important points. Trusts should ensure that GPs and hospital professionals, as well as patients, are involved in the design and content of discharge templates and that medical staff receive training that covers how to write efficiently and concisely. In order to achieve this multi-perspective design of discharge templates, future research needs to consider other perspectives on letter content, particularly those of patients. Staff training on producing high-quality discharge letters has the potential to save clinician time, reduce clinical risk, and improve clinician and patient experiences. Hence, future research should focus on developing, implementing and evaluating training interventions and improving template contents in order to increase letter quality, clinician confidence producing them, and letter comprehensibility to GPs and patients.
